# Heavy Influenza Death Roll

**Published:** 1918-12-07

**Authors:** 


### THE LONDON HOSPITAL QUARTERLY REPORT.

### Heavy Influenza Death Roll.

AT the Quarterly Court of the London Hospital, held on the 4th instant, the House Committee reported that in spite of the declaration of an Armistice, and the certainty of an entirely victorious peace at an early date, the hospital is still being worked under great difficulties, due to the fact that one result of an army of occupation being sent to Germany is that a large staff of doctors will still be required abroad, and that the War Office is therefore compelled still to call up its medical and surgical staff.

INFLUENZA EPIDEMIC.

Owing to the very serious epidemic of influenza, the House Committee had to take two floors of wards and turn them into pneumonia wards for the treatment of these patients. Since November 4 they have taken in over 400 pneumonia cases, and unfortunately 181 of them have died. The taking over of these wards has resulted in a further shortage of surgical beds. On the other hand, within a month the hospital will get back the beds which have been so long allotted to wounded officers. All this entails entire reorganisation and reallotment of the beds.

Tribute is paid to the matron for the most efficient way in which she has managed the nursing of the hospital in the face of almost impossible difficulties. With every possible bed in the hospital occupied, and 160 nurses off duty at a time, suffering from influenza, it has been a stupendous task to keep things going.

"FULL-TIME" PROFESSORS.

A Committee consisting of the Medical Council and the House Governor is now engaged in considering the question of appointing "full-time" professors in medicine, surgery, etc., but they have not yet sent the House Committee their report.

ONE MILLION HALF-CROWN APPEAL.

The House Committee report that the Chairman's appeal for a million half-crowns, to be presented to the President, Her Majesty Queen Alexandra, as a birthday gift, has been an entire success. A Committee formed by some of the prominent Danes in London and Copenhagen has been a feature of the collection, and a large sum was collected in Denmark. It is hoped that the President will come to the hospital to receive the gift in person.

ENLARGEMENT OF THE HOSPITAL.

The Committee report that the cost of running the hospital is a third more than it was even eighteen months ago. They therefore require a vastly larger sum in order to pay their way and to pay for the enlargement of the hospital which was decided upon before the war. It is absolutely essential for the efficient working of the hospital in the future that there should be large aural wards for men, women, and children as soon as possible. At present there are under twenty beds for aural cases, which is insufficient.

A hostel for the doctors is also in contemplation. Since the large increase of work necessarily entails an increase of staff, there are not sufficient bedrooms in the building to accommodate the extra doctors required.

MEDICAL RESEARCH COMMITTEE'S REPORT.

In order to test the value of the Wassermann reaction some 104 cases of patients, some of whom were known to be suffering from syphilis and some not, had samples of their blood sent to various bacteriologists in London. In the return of positive and negative results the London Hospital was right in every single case. The report complimented the London Hospital on its work throughout, and mentioned by name many of the workers there—Dr. Sequeira (who is in charge of the department), Dr. McIntosh and Dr. Fildes in Dr. Bulloch's laboratory, Dr. Turnbull and Dr. Donald in the pathological department, and Mr. Vilvandre as Dr. Sequeira's assistant.

DEATH OF MR. ROBERT WIGRAM.

The death was reported of Mr. Robert Wigram, a member of the Committee since 1894. Mr. Wigram not only attended the Committee with great regularity, but for years spent Thursday afternoons visiting the wards, where he was always greatly welcomed.

GENERAL EAST-END TRADESMEN'S DINNER.

As the result of a dinner given by the East-End Tradesmen's Association for the benefit of the hospital, the handsome sum of over £700 was contributed by the company present to the funds of the hospital. The hospital is greatly indebted to the Association for their strenuous work for the hospital over a period of fifty years.

WAR SERVICES OF 1,700 "LONDONERS."

Nearly 1,700 "Londoners" have been serving during the war—over 1,200 doctors and students in the Army and Navy and Air Force, and 229 nurses with these Forces. The rest of the number consists of members of the House Committee and lay staff. A great many distinctions have been gained by those serving, amongst them the Distinguished Service Cross with a Bar, and the Military Cross with a Second Bar.

